# Commentary: Serving the nation, serving the people: echoes of war in the early NHS

**DOI:** 10.1136/medhum-2019-011760

**Published:** 2020-06-26

**Authors:** Roberta Bivins

**Affiliations:** Centre for the History of Medicine, University of Warwick, Coventry, UK

**Keywords:** cultural history, medical humanities, popular media, politics

## Abstract

It is something of a cliché to speak of Britain as having been transformed by the traumas of World War II and by its aftermath. From the advent of the ‘cradle to grave’ Welfare State to the end of (formal) empire, the effects of total war were enduring. Typically, they have been explored in relation to demographic, socioeconomic, technological and geopolitical trends and events. Yet as the articles in this volume observe across a variety of examples, World War II affected individuals, groups and communities in ways both intimate and immediate. For them, its effects were directly *embodied*. That is, they were experienced physically and emotionally—in physical and mental wounds, in ruptured domesticities and new opportunities and in the wholesale disruption and re-formation of communities displaced by bombing and reconstruction. So it is, perhaps, unsurprising that Britain’s post-war National Health Service, as the state institution charged with managing the bodies and behaviour of the British people, was itself permeated by a ‘wartime spirit’ long after the cessation of international hostilities.

The National Health Service (NHS) has been ‘beyond the battlefield’ since its postwar inception—most healthcare for active military personnel is delivered under the auspices of the Ministry of Defence. And of course, the NHS did not spring de novo from the chaos of war. Although I cannot do justice to it here, there is an expansive literature exploring the complex relationship of the British NHS to both its immediate precursors—including the wartime Emergency Medical Service, the Highlands and Islands Medical Service, the Welsh Tredegar Medical Aid Society—and the mixed economy of welfare that preceded nationalisation, as well as growing attention to its emergence and effects as a Bellwether cultural institution (as an introduction to these subjects, readers can consult Webster 1988, 1996, vol. 1; Timmins 2001; Gorsky 2008 will guide further exploration; and Davis 2011; Hayes (2012); Bivins, 2017; Bar-Haim 2018; Mold et al 2019; Saunders 2019 offer examples of the latter approach). Instead, reflecting on this issue’s concern with the effects of war ‘beyond the battlefield’, this commentary will demonstrate through contemporary primary sources that political representations, popular understandings, visual depictions and individual experiences of the Service remain haunted by the exigencies of an embattled and rationed nation and the crisis mode of its early years.

Under continuous and very public discussion from at least the publication of the Beveridge Report in 1942 (contemporary Gallup polling indicated that some 95% of the public had heard of the Beveridge report less than a year after publication), the postwar British welfare state, and in particular the NHS, was construed rhetorically and visually as a reward for military and civilian war survivors. Editorial cartoons from the period make this point very directly. Two of the most famous are explicit: ‘O rare and refreshing Beveridge’, a *Daily Mail* cartoon from 1942, showed a grinning British soldier on the battlefield raising an overflowing Beveridge-shaped Toby jug, cheering ‘Here’s to the brave new world’. The beer’s frothy head was labelled ‘social security’ (Illingworth 1942). Wrapped around the cartoon was extensive news and editorial coverage of the benefits Beveridge proposed for ‘everyone’, described as a ‘plan to abolish want’. Proposals for universal postwar medical services featured prominently on the page.

A later Philip Zec cartoon (figure 1) for the left-leaning *Daily Mirror* newspaper in 1945 (subsequently reused in a Labour Party political pamphlet) portrayed an angry British couple demanding the benefits they had earned from a reluctant state, figured as a Churchillian grocer in a uniform labelled ‘Tory Peace Stores (very limited)’. The demobbed and disgruntled male shopper exclaimed ‘What do you mean - you’re out of stock – I’ve paid twice for these goods – once in 1914 and again in 1939!’ Under the counter and marked with the class-conscious instruction ‘the fruits of victory are reserved for the rich and privileged’ sit ‘decent schools’, ‘good homes’, ‘jobs’ and ‘proper medical attention’ (Zec 1945). The British public had become accustomed to ‘fair shares’, not least through wartime rationing, and had been promised, by Beveridge and by wartime governments, a fairer society as a reward for their fortitude and sacrifice. That equal access to ‘proper medical attention’ was self-evidently fundamental to a just society that became and remained a core value for the NHS, one that was fought for on the battlefield and Home Front and one for which many would remain willing to fight. Indeed, Abram Games’ famous and famously suppressed wartime poster featuring the revolutionary Finsbury Health Centre, ‘Your Britain. Fight for it Now’ exemplifies both this belief that modern health services would be among the rewards of victory, and elite ambivalence towards it (Games 1943; Bivins 2014; National Army Museum 2019).

**Figure 1:**
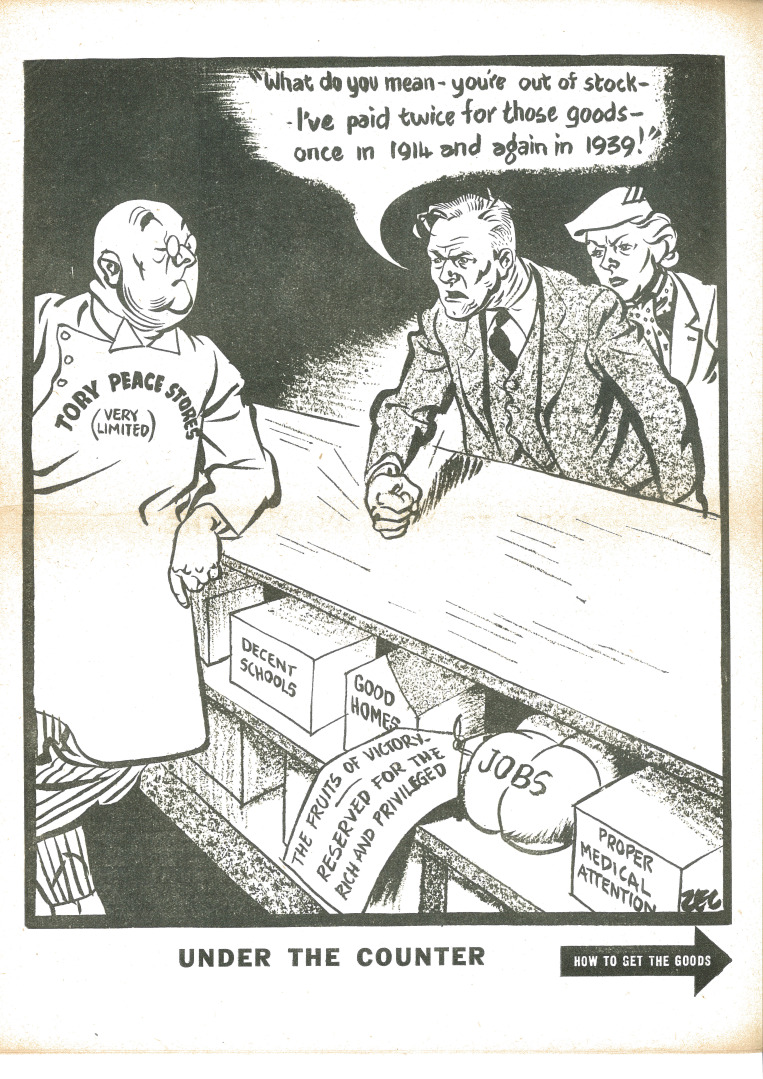
Philip Zec, ‘Under the Counter’, *Daily Mirror* 1945, reprinted in ‘Straight Left’, Labour Party pamphlet, 1945 (pg. 2). Courtesy, TUC Library Collections, London Metropolitan University.

Debates before and after the NHS Appointed Day in 1948 borrowed heavily from the language of the military crisis that had preceded it, positioning the nascent health services as a site for continued patriotic effort and even sacrifice. Proponents and critics of NHS alike made their cases by invoking duty, emergency and public service. Looking ahead to the NHS just after peace was declared in 1945, for example, government ministries described hospital staffing as ‘an urgent national need’, called for ‘thousands of recruits’ and appealed to the young in particular to ‘come and play their part in this great field of service’ (Ministry of Health, Ministry of Labour and National Service, Secretary of State for Scotland 1945, 1–2). Later, the Ministry of Health introduced the NHS through explicit analogies with the military. Describing it as ‘a service for the nation’, one early pamphlet asserted that nationalising healthcare would ‘make the total cost of the Service a Charge upon the national income *in the same way as the Defence Services* and other national necessities.’ To provide this ‘national necessity’, readers were told, required ‘the determined efforts of a people paying its way by its work’. In other words, all who benefited from the health service also owed it their own service, in the form of increased personal productivity in the workplace. While ‘the public’ were duty-bound to use the service ‘properly and economically’—drawing on a trope of sensible self-abnegation familiarised by experiences of rationing and extensive anti-waste propaganda during the war (accessible via Harris and Webb 2017)—it was the ‘duty’ of the Minister of Health to improve health and prevent illness and to ensure that all citizens were able to exercise their new ‘right to medical care’. This widely circulated document spoke, too, of ‘freely given service’ and called for ‘loyalty’ both to and from its patients and doctors (Ministry of Health and Central Office of Information c. 1951, 2, 34, 5, 14, 16, respectively).

The visible precariousness of the new services, which were in these early days also often explicitly rationed despite the rhetoric of universality, added to the sense of continuity between Britain at war and the NHS. The *National Health Service*, a 1951 leaflet created to introduce and publicise the NHS to domestic and international audiences opened by juxtaposing the Service’s ‘aim’ of addressing all health needs with stark commentary on ‘present shortages’ in staffing, infrastructure and supplies lingering after the war. Under such conditions, it admonished ‘all these services cannot all be fully provided at once’ and would therefore be provided to the most necessitous and vulnerable first (Ministry of Health and Central Office of Information c. 1951, 1, 5). Implicit rationing has remained a feature of the NHS ever since and is now institutionalised via structures including the National Institute for Health and Care Excellence.

In its first decade, the understaffed NHS faced a surge of pent-up ill health, tellingly described by a Trade Unions Council commentator as the ‘wartime arrears for treatment’ of injuries attributable to industrial production under arduous and unhealthy conditions (Hartwell 1953). To manage almost unmanageable need, the Ministry of Health and medical professionals devised what would become enduring strategies intended to limit demand, ration provision and enhance recruitment to the professions and the Service more generally. At the heart of these strategies were discourses which positioned both work in the NHS and public self-care and abstention from service use as forms of national service. Nursing recruitment posters and exhibitions represented nursing as a commitment to national service; one exhibition even figured the future nurse *as* the nation, under the slogan ‘Meet Nurse Britain’ (Central Office of Information n.d.b). Such emotive representations filtered through to NHS staff at every level. For instance, a 1962 pamphlet on food hygiene forcefully reminded NHS kitchen staff ‘Anything less than your best might cause serious harm’ (Ministry of Health 1962).

In the 1940s and 1950s, health education posters covering everything from diet to hospital cleansing and food preparation also drew on language routinised during the war: ‘Six Health Hints [to] Keep Fighting Fit’ readily transformed into ‘The Seven Rules of Health’, while ‘self-restraint’ continued to be lauded as a quietly heroic feature of the British character (respectively, Central Office of Information n.d.a.; Ministry of Health c. 1950s a; Hartwell 1953, 2). Other messages called on patients and carers to comply with health advice specifically to ‘protect the community’ and to limit their use of the NHS, asserting: ‘If the Service is to do the most good to the greatest number, care must be taken to see that it is not overtaxed by unreasonable demands’ (Ministry of Health c. 1950s b, 1950). Another leaflet, produced by the British Medical Association, echoed Home Front propaganda messages to ‘do your bit’ and ‘lend a hand’ with the slogan: ‘Everyone can help’—in this case by not bothering their doctors with trivial concerns (British Medical Association 1948). The public was also exhorted to join a variety of voluntary bodies, including the ‘National Hospital Service Reserves’, modelled on the World War II emergency services and intended to help fight the Cold War: ‘You can’t be certain – you can be ready’, one poster proclaimed (H.M. Government c. 1951). Another, aimed at promoting mental health services, actively recruited the public to its cause, again much as they had been urged to volunteer for service between 1939 and 1945: ‘there is a vital contribution to be made by the community … Without the active co-operation of the Public, no Service can be fully efficient’ (Ministry of Health and Central Office of Information 1958). And men who took up careers in understaffed and unattractive areas of nursing were even offered deferrals of National Service: here, working in the NHS was directly equated to military service (Ministry of Labour and National Service c. 1950s).

By the 1970s and 1980s, NHS propaganda that explicitly evoked the communitarianism of the war effort and postwar austerity waned in visibility, displaced by languages of risks, entitlements, means-testing and consumerism. Yet the sense of the NHS as a site for sacrifice and public service has not disappeared. If anything, it has been heightened by the re-emergence of discourses that position the NHS as a precious but threatened resource, and as a symbol of the best attributes of national character. Perhaps tellingly, warlike language has become commonplace in NHS activism, in which we see ‘battles’ to save threatened hospitals, ‘buggy armies’ defending maternity and child health services and a flood of exhortations to the public to ‘fight’ to ‘save the NHS’ (Crane 2019a, 2019b). Underpinning this metaphorical bellicosity is a familiar narrative that this is a battle to preserve or regain hard-won rights and preserve equality. And alongside this language, what imagery is used? Often, photographic images and graphic stylings evoking Bevan, the early NHS, and—whether deliberately or unintentionally—the ‘Blitz spirit’ with which the Service was infused in its wartime cradle and austere quotations (some fictional) attributed to Bevan, became a persistent trope of 2012 debates around the Health and Social Care Bill, the 2016 junior doctors’ strike and the 2017 UK general election, appearing on social media, placards, banners, graffiti, t-shirts, tote bags and even tea towels (A Better NHS 2012; Neal 2016; Anonymous 2017; Lorenz and Davies, 2017; Worthington 2017a, Worthington, 2017b; Sully, 2018).
